# Integrin **β**1–rich extracellular vesicles of kidney recruit Fn1^+^ macrophages to aggravate ischemia-reperfusion–induced inflammation

**DOI:** 10.1172/jci.insight.169885

**Published:** 2024-01-23

**Authors:** Wenjuan Wang, Xuejing Ren, Xiangmei Chen, Quan Hong, Guangyan Cai

**Affiliations:** 1 School of Medicine, Nankai University, Tianjin, China.; 2 Department of Nephrology, First Medical Center of Chinese PLA General Hospital, National Key Laboratory of Kidney Diseases, National Clinical Research Center for Kidney Diseases, Beijing Key Laboratory of Kidney Diseases Research, Beijing, China.; 3 Zhengzhou University People’s Hospital, Henan Provincial People’s Hospital, Henan Key Laboratory of Kidney Disease and Immunology, Zhengzhou, Henan, China.

**Keywords:** Nephrology, Integrins, Macrophages, Monocytes

## Abstract

Ischemia-reperfusion injury–induced (IRI-induced) acute kidney injury is accompanied by mononuclear phagocyte (MP) invasion and inflammation. However, systematic analysis of extracellular vesicle–carried (EV-carried) proteins mediating intercellular crosstalk in the IRI microenvironment is still lacking. Multiomics analysis combining single-cell RNA-Seq data of kidney and protein profiling of kidney-EV was used to elucidate the intercellular communication between proximal tubular cells (PTs) and MP. Targeted adhesion and migration of various MPs were caused by the secretion of multiple chemokines as well as integrin β1–rich EV by ischemic-damaged PTs after IRI. These recruited MPs, especially Fn1^+^ macrophagocyte, amplified the surviving PT’s inflammatory response by secreting the inflammatory factors TNF-α, MCP-1, and thrombospondin 1 (THBS-1), which could interact with integrin β1 to promote more MP adhesion and interact with surviving PT to further promote the secretion of IL-1β. However, GW4869 reduced MP infiltration and maintained a moderate inflammatory level likely by blocking EV secretion. Our findings establish the molecular bases by which chemokines and kidney-EV mediate PT-MP crosstalk in early IRI and provide insights into systematic intercellular communication.

## Abstract 2

<p>Chemokines and kidney extracellular vesicles mediate cross talk between proximal tubular cells and mononuclear phagocytes/macrophagocytes in early ischemia‒reperfusion injury.</p>

## Graphical abstract

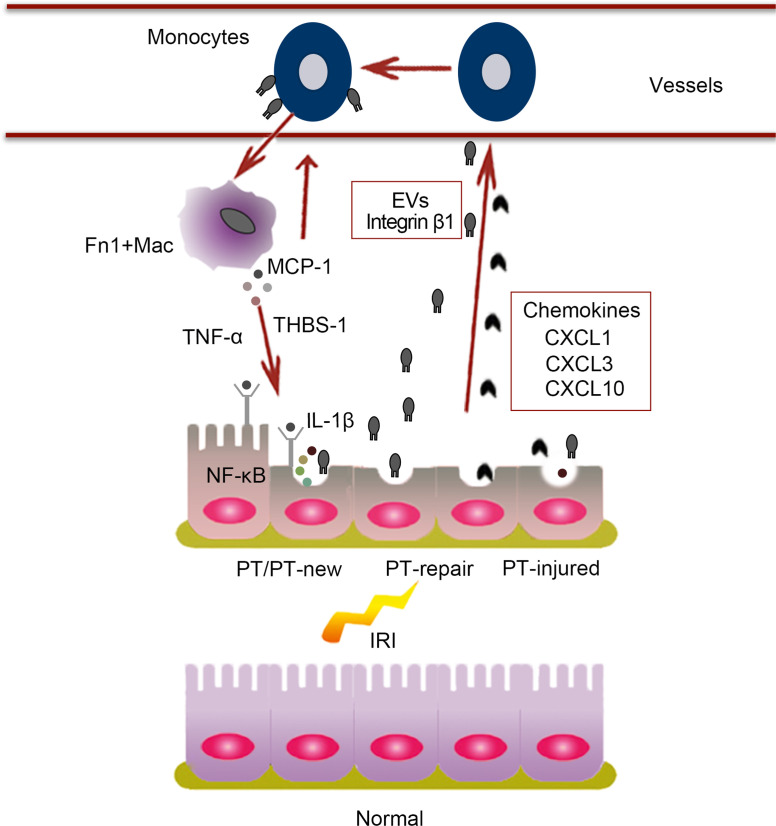

## Introduction

Acute kidney injury (AKI) is a common disease that is a serious threat to human health because of its low diagnosis rate and lack of timely treatment, which greatly increase the risk of severe AKI and chronic kidney disease (CKD) (1, 2). Ischemia-reperfusion injury (IRI) is recognized as a major cause of clinical kidney injury and is always accompanied by mononuclear phagocyte (MP) invasion and inflammation (3–6). A better understanding of the cellular pathophysiological processes underlying IRI-induced inflammation may lead to the search for novel therapeutic targets to reduce injury and prevent CKD progression.

IRI-induced AKI is mainly concentrated in proximal tubular cells (PTs) in the corticomedullary junction. Single-cell RNA-Seq (scRNA-Seq) results show that PT-injured cells had proinflammatory and profibrotic properties, which eventually led to renal pathological repair, such as renal tubule atrophy and interstitial fibrosis (6, 7). Interestingly, the corticomedullary junction with the most PT damage was accompanied by significant MP infiltration and inflammation, indicating crosstalk between both.

The number of infiltrating immune cells and the degree of the immune inflammatory response determine the outcome of AKI. Both innate and adaptive immunity are involved in the damage and repair of IRI-induced AKI (8). A spatial transcription sequencing study showed that macrophages in normal kidneys were mainly confined to areas with rich blood supply in the medulla. Once IRI occurred, peripheral blood macrophage subsets were specifically chemotactic to the corticomedullary junction after 2 hours of IRI, while in sepsis-induced IRI, these cells had a diffuse distribution, indicating that MPs played a more prominent role in the above IRI process (9–11). Specifically, at the early stage of AKI, MPs residing in the kidney and circulating in the blood are sequentially activated, releasing mononuclear chemotactic protein-1 (MCP-1), chemokine (CXC motif) ligand-1 (CXCL1), and CSF1 to recruit more MPs, which may lead to irreversible kidney damage (11–15). Furthermore, blocking MP infiltration significantly reduced the degree of kidney injury and improved the long-term prognosis (11). However, in the IRI microenvironment, whether there are other mechanisms to recruit MPs and the subsequent mechanisms to amplify renal inflammation remain unclear.

Although scRNA-Seq enables exploration of cellular interactions through receptor-ligand interaction analysis, some intercellular communications are mediated by extracellular vesicles (EV) (16, 17). EV are a class of bilayer membrane structures containing proteins, noncoding RNAs, mRNAs, and other molecules and are involved in many physiological and pathological processes, including immune regulation, mitochondrial metabolism, oxidative stress, cell damage and regeneration, tumorigenesis, migration, and delayed senescence (18–24). At present, most studies have mainly focused on the roles of EV from cell cultures or body fluids. There have been few studies on tissue-derived EV in the specific tissue microenvironment of IRI-AKI, and even fewer studies have used multiomics analysis of scRNA-Seq data and tissue-derived EV protein profiles to comprehensively examine intercellular crosstalk between PTs and MPs during AKI.

Therefore, this study examined the intercellular crosstalk between PTs and MPs by combining scRNA-Seq data and tissue EV protein profiles for multiomics analysis to systematically elucidate the change and mechanism of the excessive inflammatory response in the IRI-induced AKI microenvironment to provide a theoretical basis for the subsequent treatment of AKI.

## Results

### Induction of AKI by IRI.

**Figure 1:**
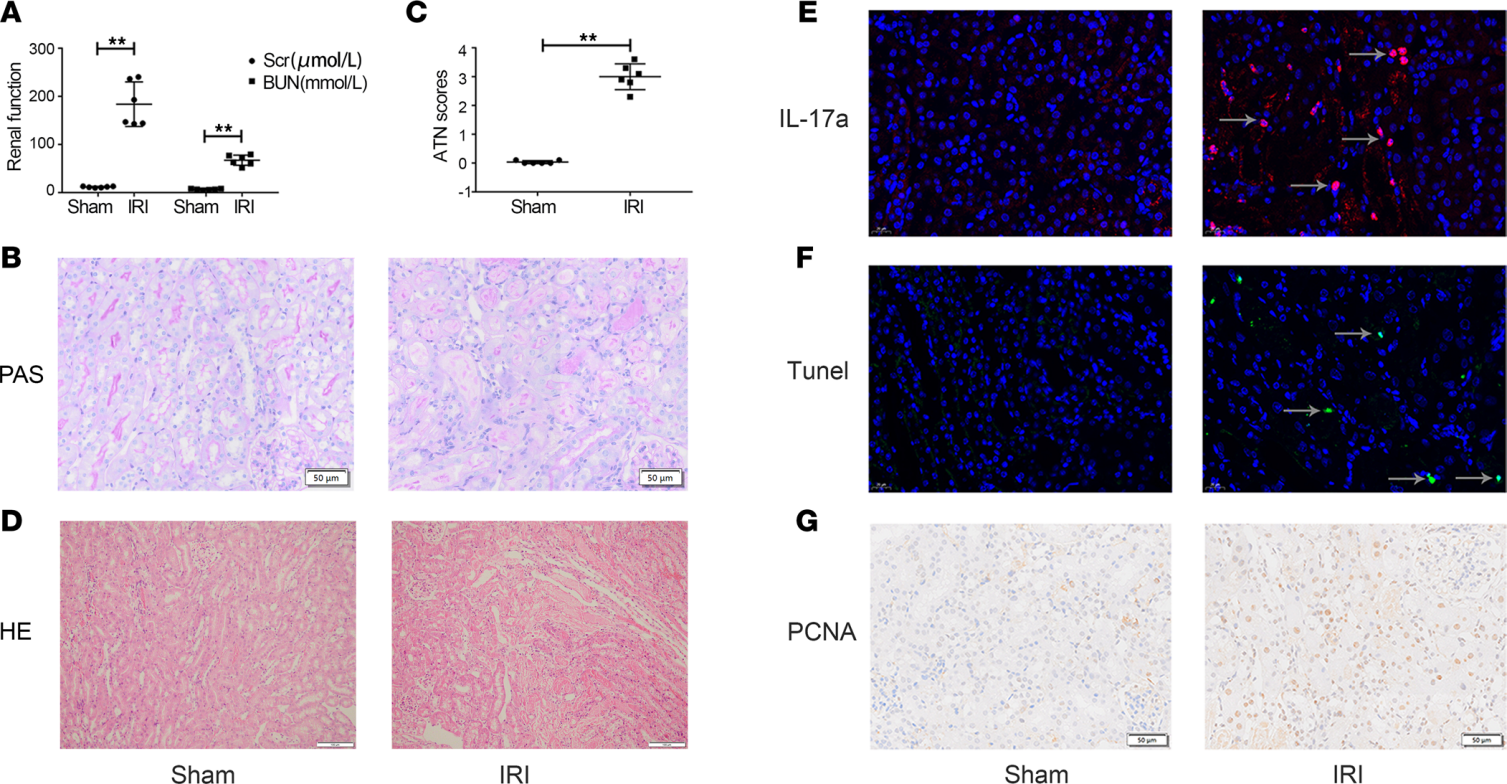
Renal response to IRI at 1 day. (**A**) Biochemical detection of representative renal function indicators (serum creatinine [Scr] and urea nitrogen [BUN]). (**B**) Periodic acid–Schiff (PAS) staining. (**C**) Acute tubular necrosis (ATN) scores. (**D**) H&E staining showing that the immune cells were characterized by blue nuclear staining and a pink-to-peach cytoplasm. (**E**) Immunofluorescence staining of IL-17a (red). (**F**) TUNEL (green) staining. (**G**) Immunohistochemical staining of renal proliferating cell nuclear antigen (PCNA, brown). *n* = 6. Data are expressed as the mean ± SD. Student’s *t* test was used for comparisons of 2 groups. **P < 0.01. Scale bars: 50 μm (**B** and **G**), 100 μm (**D**), and 20 μm (**E** and **F**).

To evaluate the degree of kidney injury at 24 hours after IRI, changes in renal function and pathological injury were detected. The results showed that the levels of serum creatinine (Scr) and urea nitrogen (BUN) were significantly increased in the IRI group compared with the sham group (Figure 1A). Periodic acid–Schiff (PAS) staining showed that injury sites in IRI kidneys — including sites of granular or vascular degeneration, dissolution, necrosis and shedding of renal tubular epithelial cells, and the formation of much proteins and/or cell casts — were mainly concentrated in PTs, which is consistent with the changing trend of acute tubular necrosis (ATN) scores (Figure 1, B and C). H&E staining shows significant inflammatory cell infiltration in the interstitial region (Figure 1D). Similarly, the expression levels of the inflammatory and profibrotic factor IL-17a, TUNEL staining, and proliferating cell nuclear antigen (PCNA) were significantly increased in IRI kidneys compared with sham kidneys (Figure 1, E–G). These results indicate that, during IRI, necrosis and apoptosis occur and are accompanied by activation of the inflammatory response and cell proliferation, which jointly participate in injury repair during IRI.

### Renal single-cell transcriptomic profiling of IRI-AKI.

**Figure 2:**
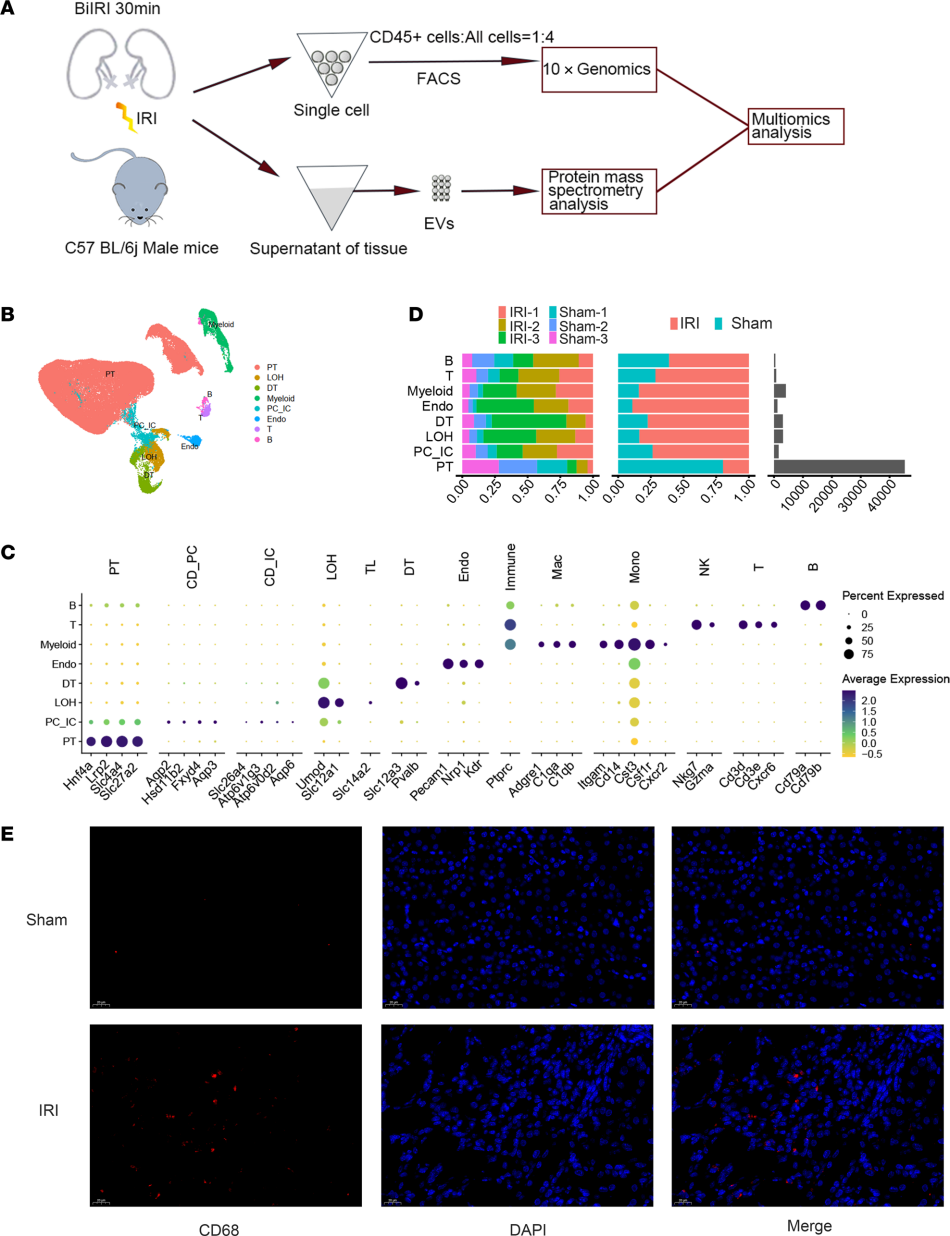
Single-cell transcriptomic profiling of the IRI kidney. (**A**) Flow chart showing the experimental design. *n* = 3. (**B**) UMAP plot showing the clustering of 59,528 cells by cell type. (**C**) Dot plot showing the representative marker genes of the 8 clusters. (**D**) Bar plot showing the comparisons of the relative proportion of the respective clusters from **B**. (**E**) Immunofluorescence staining of CD68 (red) in kidney tissue. Scale bar: 20 μm. BiIRI, Bbilateral ischemia reperfusion injury; EV, extracellular vesicle; PT, proximal tubular cell; LOH, loop of Henle; DT, distal tubule cell; PC_IC, principal and intercalated collecting duct compartment; Endo, endotheliocyte; T, T cell; B, B cell; mono, monocyte; mac, macrophage.

The overlap of the PT-injured cells and immune cell distribution in the above results indicate crosstalk between them. To understand the underlying mechanism, we first obtained a single-cell suspension from the fresh kidneys of WT C57BL/6 mice at 1 day after sham or IRI surgery. A 1:4 mixture of CD45+ cells sorted by FACS and all isolated single cells was subjected to scRNA-Seq analysis by 10× Genomics. Kidney tissue was enzymatically hydrolyzed to obtain tissue supernatant to extract EV, and proteins extracted from the isolated EV were subjected to mass spectrometry (MS) analysis. Finally, we systematically elucidated intercellular crosstalk through multiomics analysis, as shown in Figure 2A.

To develop a comprehensive profile of the cellular and molecular changes, the transcriptomes of 59,528 high-quality cells passed quality control (Supplemental Figure 1; supplemental material available online with this article; https://doi.org/10.1172/jci.insight.169885DS1). Renal cell populations were identified using dimension reduction analysis. Uniform manifold approximation and projection (UMAP) analysis revealed cell clusters, including PTs, loop of Henle (LOH) cells, distal tubule cells (DTs), myeloid cells, principal and intercalated collecting duct compartment (PC_IC) cells, endothelial cells (Endos), T cells, and B cells (Figure 2B). Figure 2C shows the cell cluster markers for each subpopulation. Among the intrinsic cells of the kidney, normal PTs accounted for 92.9% of all sequenced cells in the sham group, but the proportion of normal PTs was 43.7% in the IRI group (Figure 2D and Table 1). Among immune cells, myeloid cells accounted for the largest proportion, and their proportion was increased 11-fold after IRI (Figure 2D and Table 1); this was confirmed by immunofluorescence staining of CD68, a marker of macrophages (Figure 2E). These results indicate that myeloid cells are the main cells with the most significant proportion change in immune cell infiltration induced by IRI.

### PT responses to IRI.

**Figure 3:**
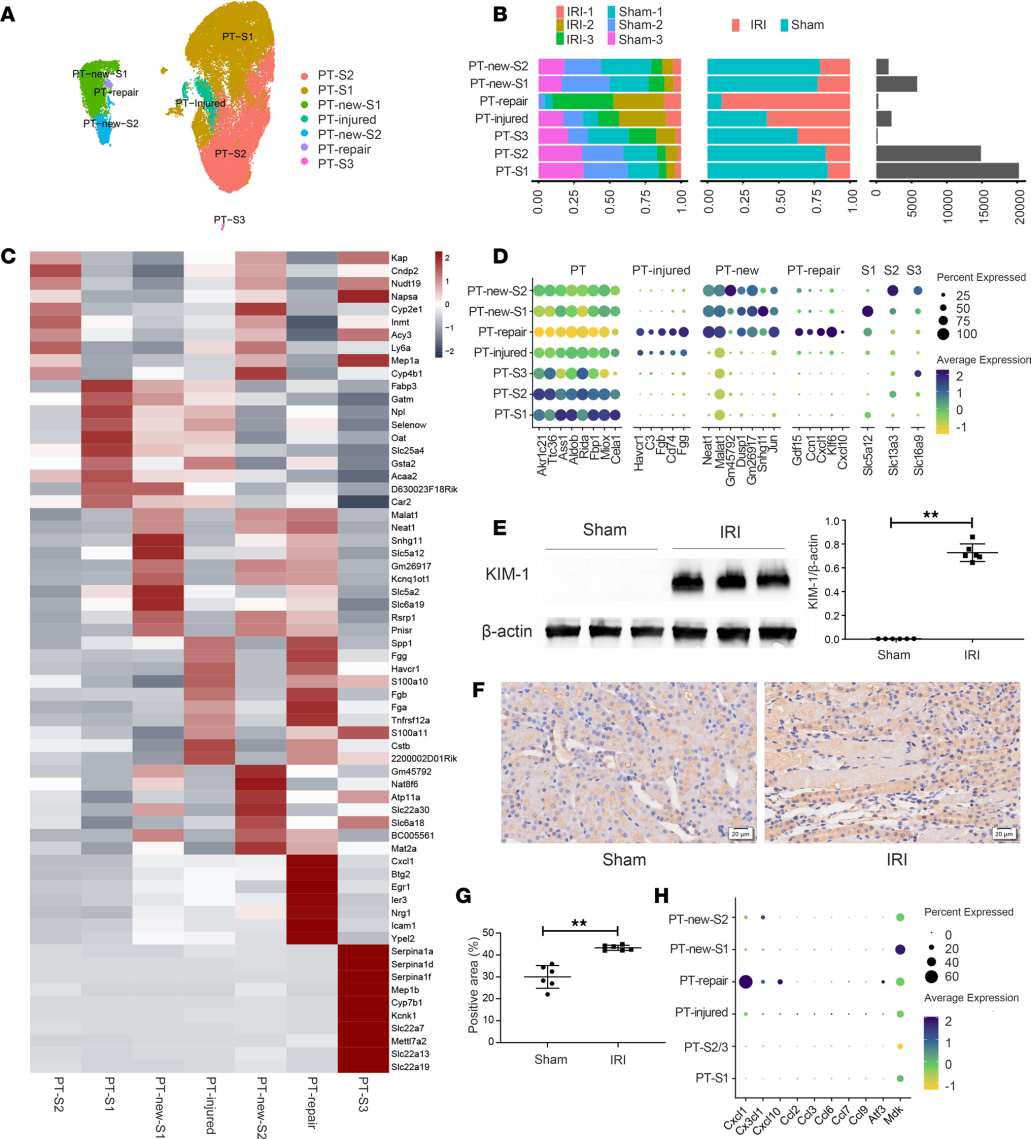
The diversity of PTs in IRI-AKI. (**A**) UMAP plot showing 7 clusters of PTs. (**B**) Bar graphs showing the relative frequencies of the respective clusters from **A**. (**C**) Heatmap showing the expression of the top 10 genes in each subpopulation of PTs. (**D**) Dot plot showing the representative marker gene of the 7 clusters. (**E**) Western blotting showing the level of KIM-1 expression. (**F**) IHC staining of integrin β1 (brown). Scale bars: 20 μm. (**G**) Quantification of integrin β1 expression. (**H**) Dot plot showing the ability of each PT subgroup to secrete chemokines. *n* = 6. Data are expressed as the mean ± SD. Student’s *t* test was used for comparisons of 2 groups. **P < 0.01.

We focused our analysis on PTs since this population suffered the most severe injury due to their specific blood supply characteristics and high metabolic activity. According to the UMAP plot, PTs showed diversity and were divided into normal PT of S1 segment (PT-S1), PT-S2, and PT-S3 cells; PT-injured; PTs associated with repair (PT-repair cells); and PT-new-S1 and PT-new-S2 cells (Figure 3A). Compared with the sham group, the AKI model group exhibited 30-fold and 5.5-fold increases in PT-repair and PT-injured cells, respectively, but significantly fewer normal PTs (Figure 3B). The top 10 genes for clustering are listed in Figure 3C. PT-new cells specifically expressed *neat1*, *Gm45792*, and *Snhg11* (Figure 3D), and these cells might well be in a transitional state between normal and damaged PTs. In addition, PT-injured cells expressed the classic marker KIM-1 and integrin β1 (Figure 3, E–G). The newly emerged group of PT repair cells specifically highly expressed *Gdf15*, *Ccn*1, *Cxcl1*, and *Klf6*, which are closely related to repair (Figure 3, D and H) (25). Compared with other PT subgroups, PT-repair cells expressed more chemokines, including *Cxcl1*, *Cx3cl1*, *Cxcl10*, and *Atf3* (Figure 3H). However, PT-injured cells exhibited increased expression of *Ccl2*, *Ccl3*, and *Ccl6* (Figure 3H). The above secreted chemokines could recruit more infiltrating immune cells, leading to irreversible inflammatory damage involved in renal fibrosis in AKI-CKD progression. In conclusion, there are various types of PTs during IRI, among which PT-injured and PT-repair cells are important in activating and amplifying subsequent immune inflammatory responses.

### Dynamic functional plasticity of myeloid cells.

**Figure 4:**
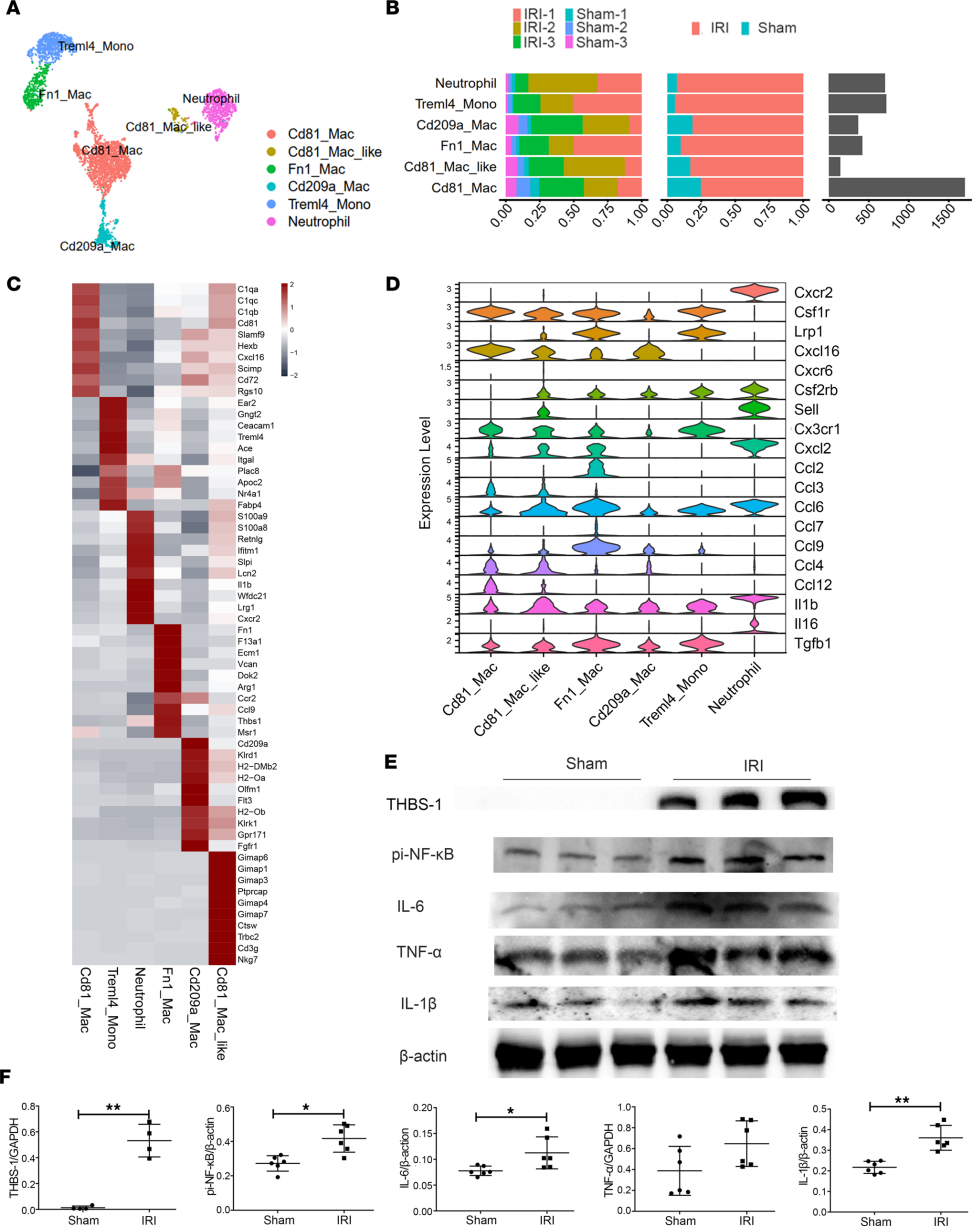
Identification of myeloid cell subtypes in IRI-AKI. (**A**) Subclustering of myeloid cells from sham- and IRI-AKI kidneys. (**B**) Bar graphs showing the proportions of the abovementioned myeloid cell subsets. (**C**) Expression of the top 10 genes in each subpopulation. (**D**) Violin diagram showing some chemokines and proinflammatory cytokines secreted by myeloid cells. (**E** and **F**) Western blotting and semiquantitative analysis showing the expression of the Fn1^+^ macrophage marker THBS-1, the proinflammatory transcription factor pi–NF-κB, and the inflammatory cytokines IL-6, TNF-α, and IL-1β. *n* = 4–6. Data are expressed as the mean ± SD. Student’s *t* test was used for comparisons of 2 groups. *P < 0.05, **P < 0.01.

To better understand the contributions of the various myeloid cells, the MPs were divided into Cd81_Mac, Cd81_like_Mac, Fn1_Mac, Cd209a_Mac, Treml4_Mono, and Neutrophil (Figure 4A). Compared with that in the sham group, the proportion of myeloid cells in the AKI group was significantly increased, and, among these increased myeloid cells, the proportion of kidney-resident Cd81_Mac was the largest (Figure 4B). Fn1_Mac highly and specifically expressed *Fn1*, *Arg1*, *Ccr2*, *Ccl9*, and *Thbs1*; the top 10 genes that were highly expressed in other groups are shown in Figure 4, C, E, and F, and Supplemental Figure 2.

Next, the biological functions of these cells were examined. Supplemental Figure 3 shows the top 50 functions and pathways with the most notable changes between different subgroups. Based on the biological process (BP) terms, Fn1_Mac was involved in neutrophil activation and degranulation, promoted mast cell differentiation, and participated in the processing and presentation of plasmacytoid DC antigens (Supplemental Figure 3A). According to Reactome analysis, Fn1_Mac was primarily involved in inflammation pathways, including scavenging by class A receptors, complement activation of C3/5, and trafficking and processing of endosomal TLR and metabolism-related pathways, including CAM PDE1 activation, HDL remodeling, and synthesis of eicosatetraenoic acid and its derivatives (Supplemental Figure 3B).

Next, we analyzed their ability to secrete chemokines (Figure 4D). Neutrophils mainly expressed *Cxcl2*, *Ccl6*, and the inflammatory factor *IL-1β*. Treml4_Mono and Fn1_Mac expressed a variety of similar chemokines, such as *Csf1r*, *Lrp1*, *Ccl6*, and *Tgfb1*, and the latter protein promoted early repair but was involved in renal fibrosis during the progression of AKI-CKD. In addition, Treml4_Mono specifically expressed *Cx3cr1*, Fn1_Mac specifically expressed *Ccl2* and *Ccl9*, and CD81_Mac specifically expressed *Csf1r* and *Lrp1* to recruit more infiltrating immune cells to amplify inflammation (Figure 4D). Finally, we detected the expression levels of the proinflammatory transcription factor pi–NF-κB and the inflammatory factors IL-6, TNF-α, and IL-1β. Compared with those in the sham group, their levels were significantly increased in the IRI group (Figure 4, E and F).

Thus, myeloid cells in the different subgroups play a variety of roles by expressing different cytokines and recruiting more infiltrating immune cells during the immune inflammatory response induced by IRI.

### Intercellular interactions between PTs and myeloid cells.

**Figure 5:**
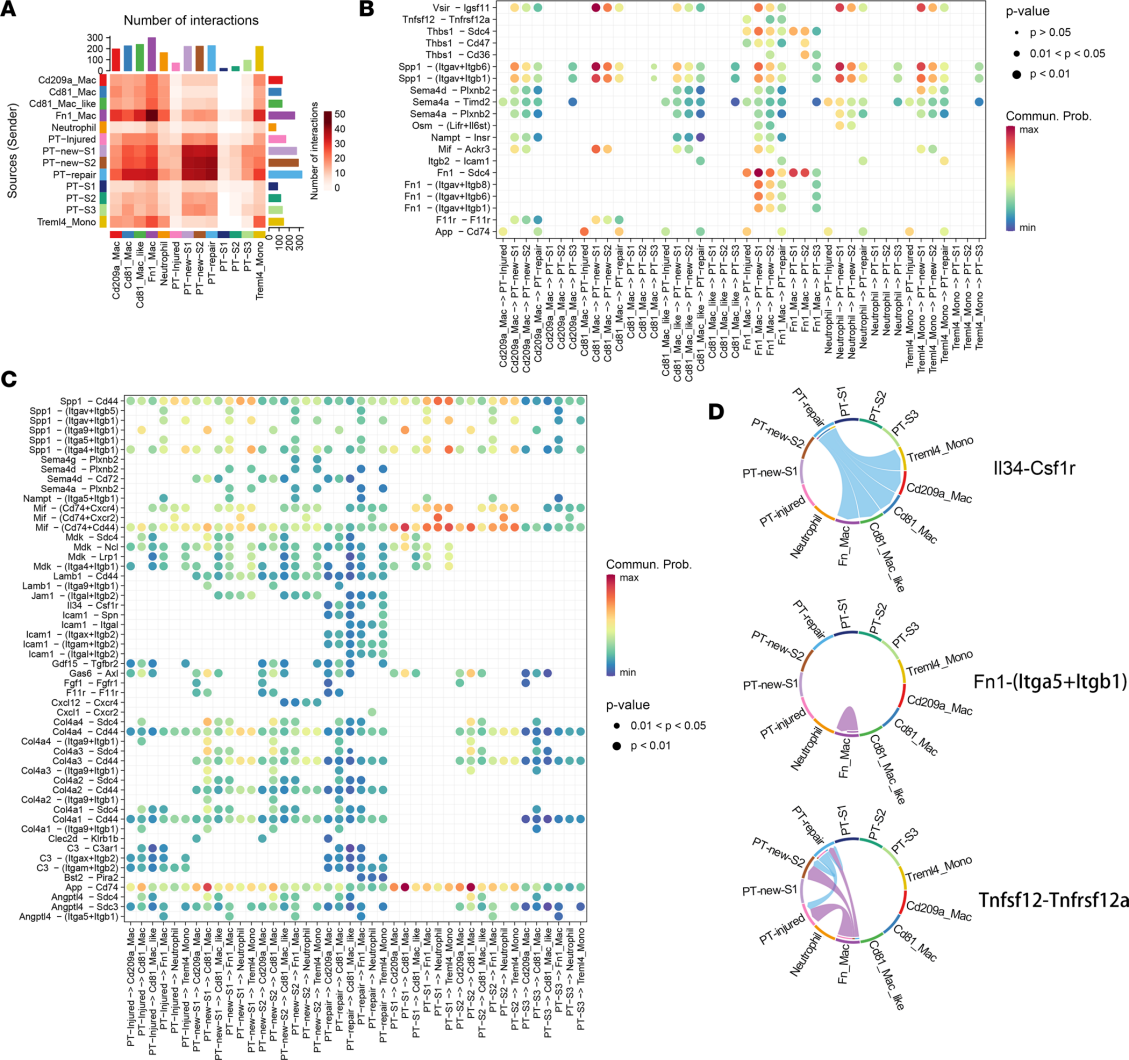
Intercellular crosstalk analysis of PTs and myeloid cells. (**A**) Heatmap showing inferred interaction numbers between each subpopulation. (**B** and **C**) The ligand-receptor interaction pairs between PTs and myeloid cells. (**D**) Chord diagram showing the myeloid cell–PT immune interaction networks.

To globally examine chemotactic signaling across different cell types, we determined the ligand-interaction numbers for all cell types (Figure 5A). There was a strong intercellular interaction between PT-injured/repair cells and MPs at the genetic level. Next, how IRI affected intercellular communication was examined using ligand-to-receptor analysis (Figure 5, B and C). PT-repair cells had increased levels of many chemokines, specifically CXCL1 (Supplemental Figure 4). Furthermore, we found that the *Cxcl1* receptor–encoding gene *Cxcr2* was mainly expressed in neutrophils, suggesting possible pathological tubular-to-immune crosstalk (Figure 5C and Supplemental Figure 4). In addition, *C3-C3ar1* and *Il34-Csf1r* might also play significant roles in these processes (Figure 5, C and D, and Supplemental Figure 4). *Angptl4 –* (*Itga5 + Itgb1*) might mediate the recruitment of Fn1_Mac by PT-injured cells (Figure 5C). In particular, *Fn1 –* (*Itga5 + Itgb1*) might promote the recruitment and migration of its own subpopulation (Figure 5D). Finally, Fn1_Mac might act on surviving PTs, including PT-repair cells, via *Fn1 –* (*ItgaV + Itgb1/6/8*), highlighting the importance of integrins (Figure 5B). Moreover, PT repair cells and Fn1_Mac might also be involved in proinflammatory reactions through the *Tnfsf12-Tnfrsf12a* interaction and fibrosis through *Tgfb1 –* (*Tgfbr1 + Tgfbr2*) (Figure 5, B and D, and Supplemental Figure 4). However, the possibility that another form of cell-to-cell communication could be mediated by EV in the IRI microenvironment should not be ignored.

### Unique protein signature of EV derived from the kidneys of IRI mice.

**Figure 6:**
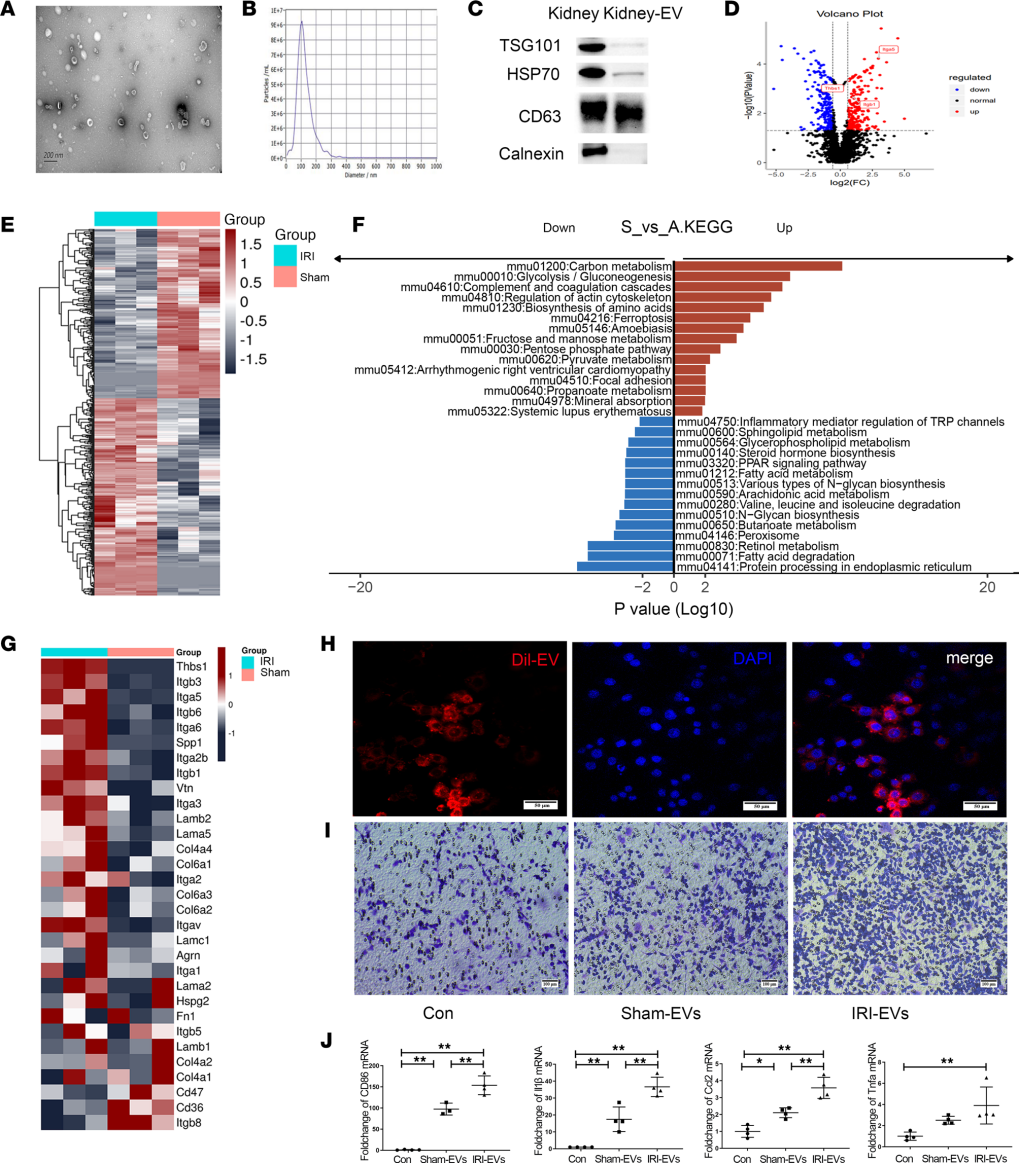
Unique protein profile and functional identification of EV isolated from sham and IRI kidneys. (**A**–**C**) Identification of EV by electron microscopy (**A**), nanoparticle tracking analysis (NTA) (**B**), and Western blotting (**C**). (**D**) Volcano plot showing the differential protein expression of IRI-EV compared with sham-EV. (**E**) Heatmap showing cluster analysis of differentially expressed proteins in EV isolated from sham versus IRI kidneys. (**F**) KEGG pathway analysis of differential proteins in EV. (**G**) Heatmap showing the typical molecules involved in extracellular matrix–receptor (ECM-receptor) interactions. (**H**) Cell internalization of Dil-labeled EV (red). (**I**) Detection of cell migration ability after stimulation with sham-EV and IRI-EV. (**J**) The expression of *CD86*, *Tnfa*, *Il1b*, and *Ccl2* detected by PCR after MPs had taken up tissue-derived EV. *n* = 3–4. Data are expressed as the mean ± SD. One-way ANOVA was used for comparisons of 3 or more groups. *P < 0.05, **P < 0.01. Scale bars: 50 μm (**H**) and 100 μm (**I**).

EV represent an important medium for intercellular crosstalk, but there have been few studies on the effect of kidney tissue–derived EV (kidney-EV) in the IRI microenvironment. Thus, we wished to determine the contribution of kidney-EV to mediating the immune response in IRI kidneys. First, kidney-EV were characterized. The cup membrane structure of EV is shown in electron micrographs (Figure 6A). The diameters of the EV ranged from 50 to 200 nm, indicating that the majority of them were exosomes (Figure 6B). Western blotting showed that kidney-EV were rich in classic markers of EV, such as CD63, TSG101, and HSP70, but did not express the cellular marker calnexin (Figure 6C).

To identify the protein cargo and function of EV, MS was performed to analyze equal amounts of protein from the kidneys of IRI and sham mice. Proteomic analysis identified a total of 2,602 proteins. Among them, 479 proteins in the cargo of EV isolated from IRI mice (IRI-EV) were differentially regulated or not detected in the cargo of EV from sham mice (sham-EV). A volcano plot was generated to identify changes between the protein expression levels in IRI- and sham-EV (Figure 6D). Among them, integrin β1 (ITGB1), integrin α5 (ITGA5), and thrombospondin 1 (THBS-1) were significantly upregulated and were mainly involved in cell recruitment and migration and in the inflammatory response. A heatmap shows quantitative changes, and many proteins were upregulated and downregulated in IRI-EV compared with sham-EV (Figure 6E). Most of these proteins were involved in wound healing, cell-substrate adhesion, and leukocyte migration (Supplemental Figure 5). The upregulated proteins of EV were involved in integrin binding, cell adhesion molecule binding, and extracellular matrix binding (Supplemental Figure 5). According to KEGG analysis, the upregulated proteins not only participated in carbon metabolism and glycolysis/gluconeogenesis, but they were also involved in the activation of complement and coagulation cascades (Figure 6F). In more detail, we displayed EV proteins involved in the ECM-receptor interaction (Figure 6G). In addition to THBS-1 and multiple integrins, including ITGA5 and ITGB1, the differentially expressed proteins involved in adhesion and migration between cells and the extracellular matrix included Spp1, which was highly expressed in EV (Figure 6G).

To simulate the function of kidney-EV in vitro, sham- and IRI-EV were endocytosed by RAW264.7 cells in vitro (Figure 6H). Cell migration ability was examined by Transwell detection. Compared with that in the control group, the number of cells that migrated to the lower chamber in the sham-EV group was increased 1.3-fold, and the number of cells that migrated to the lower chamber in the IRI-EV group was further increased (Figure 6I). Next, PCR was used to examine molecular function changes in MPs that had taken up kidney-EV. Compared with that in the control group, the expression of *Cd86*, *Tnfa*, *Il1b*, and *Ccl2* was increased in the sham group and further increased in the IRI group (Figure 6J). In addition, the differentiation of RAW264.7 cells into Fn1^+^ macrophages in the tissue microenvironment simulated by the dissociated fluid of sham- and IRI-kidney tissues was examined by flow cytometry by detecting the expression of arginase-1 (Arg-1), a top 6 marker gene of Fn1^+^ macrophages (Figure 4C). The Arg-1 expression level in RAW264.7 cells induced by the tissue microenvironment in the IRI group was significantly higher than that in the sham group (Supplemental Figure 6).

We concluded that renal tubular epithelial cells suffering from IRI secreted integrin β1–rich EV to promote the recruitment and migration of MPs, especially Fn1_Mac, as well as to activate their proinflammatory properties, participating in the activation of immune cells and the cascade amplification reaction of inflammation in the IRI microenvironment.

### Cell subpopulation tracing and the function of proteins in EV.

**Figure 7:**
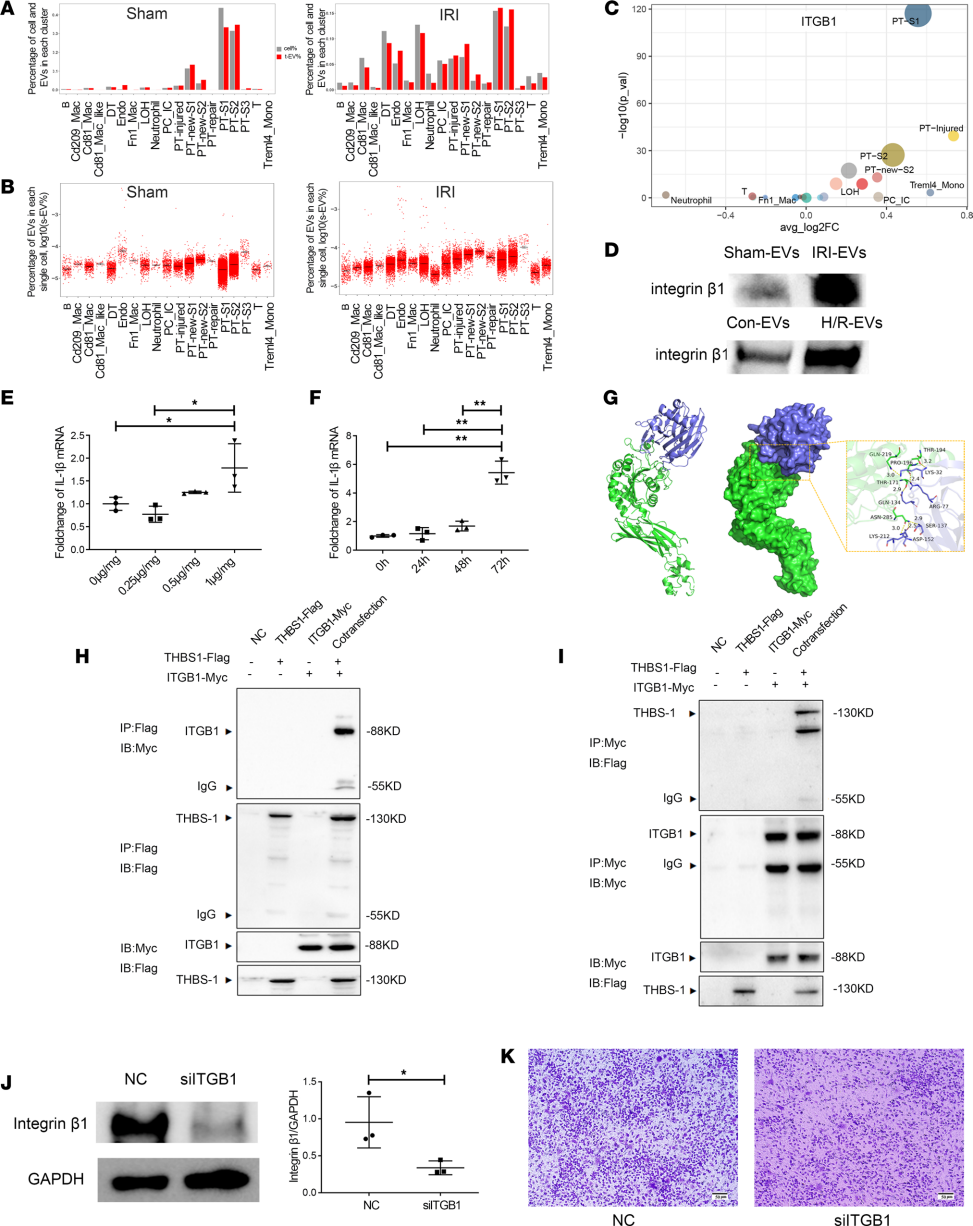
Traceability and mechanisms of proteins in tissue-derived EV. (**A**) Percentage of cells of each cluster among the total cells (cell%) and percentage of EV from each cluster (t-EV%) in the sham and IRI groups. (**B**) Percentage of EV (s-EV%) from each single cell. (**C**) Dot plot showing the expression of *ITGB1* in each subgroup. (**D**) Western blotting showing the production of integrin β1 in kidney-EV of IRI and HK2 cell–EV of hypoxic reoxygenation (H/R). (**E** and **F**) Bar plot showing the expression of IL-1β in HK2 cells exposed to H/R after stimulation with THBS-1 at different concentrations and for different times. (**G**) Protein molecular docking of THBS-1 (slate) and integrin β1 (green). The detailed binding mode of integrin β1 with THBS-1 is also presented with hydrogen bonds (yellow dashes). (**H** and **I**) The interaction between THBS-1 and integrin β1 revealed by coimmunoprecipitation (co-IP). (**J**) Western blotting showing the silencing effect of siITGB1. (**K**) Effect of siITGB1-treated HK2 cells on MP migration. *n* = 3. Scale bars: 50 μm. Data are expressed as the mean ± SD. Student’s *t* test was used for comparisons of 2 groups (**J**), and 1-way ANOVA was used for comparisons of 3 or more groups (**E** and **F**). *P < 0.05, **P < 0.01.

To assess the cell types that secrete EV proteins mediating cell migration, multiomics analysis by combining the EV protein profile with scRNA-Seq data was performed to trace the origin of EV proteins. In the sham group, mainly normal S1 and S2 PTs secreted EV (Figure 7A). In the IRI group, the abilities of the newly emerged PT subgroups — especially the PT-injured, PT-new-S1/2, PT-repair, DT, endo, and LOH subgroups — to secrete EV after injury were significantly elevated, indicating that EV proteins were involved in the subsequent injury and repair process (Figure 7A). Then, the individual abilities of single cells to release EV were evaluated. The results show that single PT-S1 and PT-S2 cells released the most EV in sham kidneys, whereas single MPs, endotheliocytes, and PTs, especially PT-repair and PT-injured cells, acquired the ability to secrete more EV in IRI kidneys (Figure 7B). More specifically, after IRI, the change in integrin β1 expression in PT-injured cells was the most significant, and it was also increased in PT-S1, PT-S2, PT-new-S2, and LOH, indicating that IRI induced integrin β1 secretion by PT with/without obvious injury (Figure 7C). Next, the production of integrin β1 in tissue-EV and cell-EV was detected. Compared with that in sham-EV, the expression of integrin β1 in IRI-EV was significantly increased (Figure 7D). Consistent with the in vivo results, the expression of integrin β1 in EV extracted from the cell culture supernatant of HK2 cells was significantly increased after H12R48-induced injury in vitro (Figure 7D). However, the expression of integrin α5 and THBS-1 showed no significant change (Supplemental Figure 7).

According to existing studies, *Cd36*, *Cd47*, and *Sdc4* are the classical receptors of *Thbs1* (26–29). In this study, *Cd36* was mainly expressed in PT-S2/S3 and some MPs, while both *Cd47* and *Sdc4* were not specific among individual cell subsets (Supplemental Figure 8). To examine the effect of THBS-1 on PTs in the IRI microenvironment, we used different concentrations of THBS-1 to stimulate HK2 cells undergoing hypoxia/reoxygenation (H/R) injury and further stimulated the cells for different times. The results show that THBS-1 promoted the expression of the inflammatory factor *Il1b* in a concentration-dependent and time-dependent manner (Figure 7, E and F). In addition, primary human renal proximal tubule cells (HPTCs) were stimulated with 1 μg/μL THBS-1, and the expression of IL-1β and TNF-α in the H/R + THBS-1 group was greater than that in the H/R group (Supplemental Figure 9).

To further clarify whether THBS-1 and integrin β1 jointly participated in cell adhesion and migration, we used molecular docking to simulate molecular binding sites (Figure 7G). The binding free energy was –222.89 kcal/mol, indicating good surface matching and a stable combination (Figure 7G). Further protein coimmunoprecipitation (co-IP) experiments show that THBS-1 interacted with integrin β1 to participate in cell migration and adhesion (Figure 7, H and I). However, once *ITGB1* was silenced by siRNA in HK2 cells, the ratio of cell migration induced by H/R-injured HK2 cells was reduced by approximately 20% (Figure 7, J and K).

These results demonstrated that, after IRI, PTs secreted more integrin β1–enriched EV, which interacted with THBS-1 and other integrins to promote the migration of immune cells to the site of injury. Furthermore, these recruited Fn1_Mac that specifically expressed THBS-1 might interact with surviving PTs by THBS-1–CD36 pairs to activate IL-1β production, exacerbating local renal inflammatory levels.

### Renal protection of GW4869 on kidney in early IRI.

**Figure 8:**
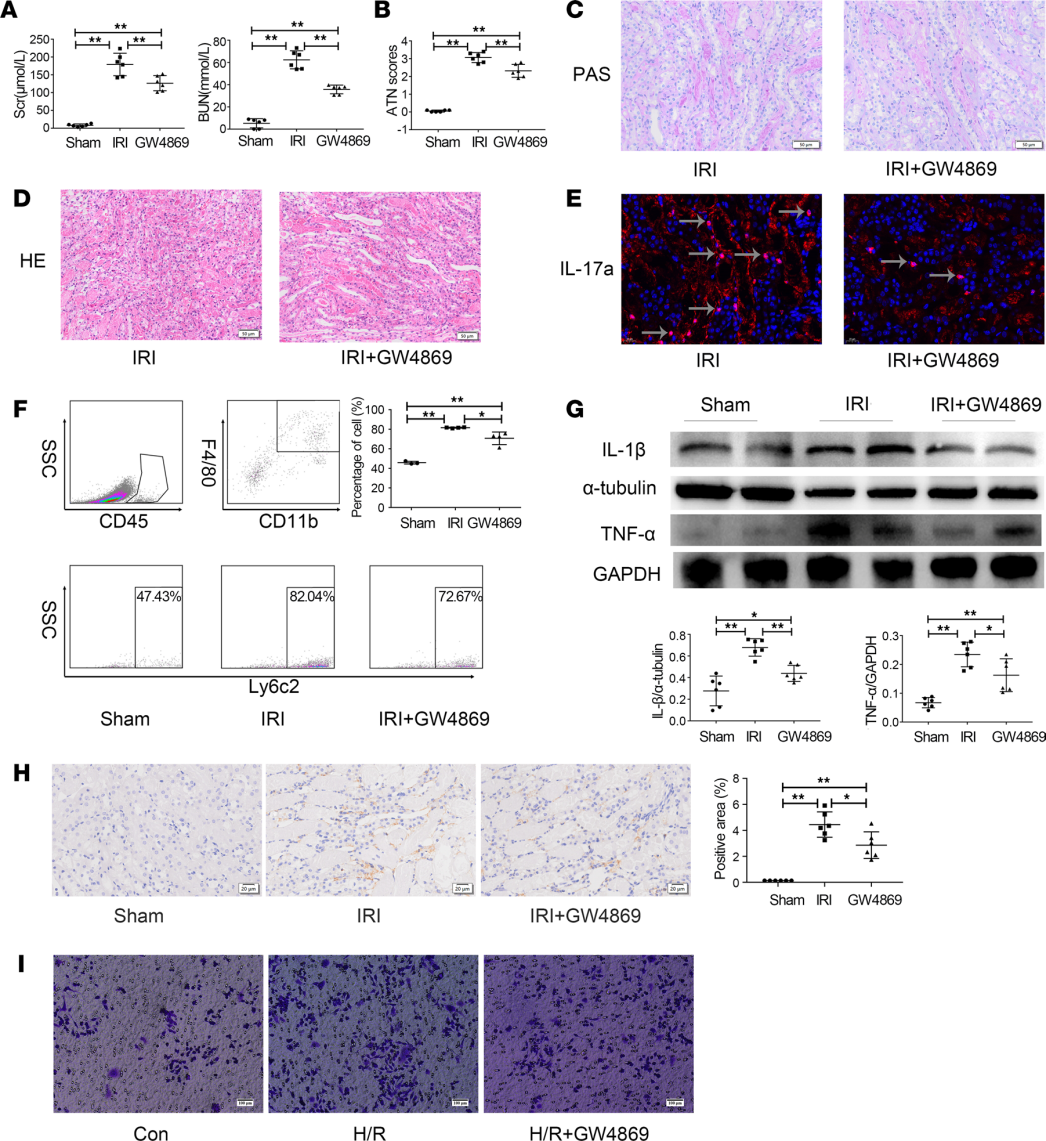
Effect of inhibiting EV secretion with GW4869 on immune cell infiltration and inflammation. (**A**) The levels of Scr and BUN. (**B**) The ATN scores. (**C** and **D**) PAS and H&E staining. (**E**) Representative immunofluorescent images of IL-17a (red). (**F**) Flow cytometry showing the proportions of F4/80^+^/CD11b^+^ macrophages in the IRI and IRI + GW4869 group. (**G**) Western blotting of the proinflammatory cytokines IL-1β and TNF-α in the sham, IRI, and IRI + GW4869 groups. (**H**) IHC analysis of THBS-1 in the sham, IRI, and IRI + GW4869 groups. (**I**) Effect of GW4869 on cell migration stimulated by HK2 cells after H/R injury. *n* = 3–6. Scale bars: 50 μm (**C** and **D**), 20 μm (**H**), and 100 μm (**I**). Data are expressed as the mean ± SD. One-way ANOVA was used for comparisons of 3 or more groups. *P < 0.05, **P < 0.01.

To further verify the role of EV in recruiting MPs in vivo, GW4869 was injected i.p. 1 hour before IRI to block EV secretion. Blocking EV with GW4869 reduced the levels of Scr and BUN by 29.4% and 42.8%, respectively (Figure 8A). The ATN scores and PAS staining results show that renal tubular epithelial cell necrosis, lumen dilation, and cast formation were reduced by 24.5% in the GW4869 group compared with the sham group (Figure 8, B and C). H&E staining revealed a corresponding decrease in immune cell infiltration (Figure 8D). The immunofluorescence result demonstrated that the expression of IL-17a was significantly reduced (Figure 8E). Flow cytometry demonstrated that the proportion of CD11b^+^/F4/80^+^ macrophages expressing the peripheral blood infiltration marker Ly6c2 increased 1.8-fold after IRI and decreased 13.3% after EV blockade (Figure 8F). Western blotting was used to further measure the expression of the inflammatory factors IL-1β and TNF-α. Compared with those in the sham group, the expression levels of IL-1β and TNF-α were significantly increased by approximately 3-fold in the IRI group but were decreased by 35.4% and 30.7%, respectively after GW4869 administration (Figure 8G). Immunostaining result showed that GW4869 significantly reduced the expression of Fn1^+^ macrophage-specific marker THBS-1 induced by IRI (Figure 8H). Finally, an in vitro coculture experiment showed that GW4869 reduced cell migration, which is induced by HK2 cells subjected to H/R injury (Figure 8I). In conclusion, GW4869 reduced inflammatory cell infiltration associated with kidney injury likely by blocking EV secretion, maintaining a moderate level of inflammation to ensure effective repair.

## Discussion

This study is the first to our knowledge to combine scRNA-Seq data and the protein profile of kidney-EV in multiomics analysis. It comprehensively elucidated the intercellular communication mediated by chemokines and EV between PTs and MPs in the early stage of IRI. Moreover, the mechanism of early activation and cascade amplification of immune inflammation in IRI was further examined.

Myeloid cells are important immune cells involved at the early stage of IRI and the progression of renal fibrosis. In this study, scRNA-Seq was used to identify a PT-repair subgroup with proinflammatory properties that recruited peripheral blood myeloid cells to the corticomedullary junction with the most severe ischemic injury by secreting CXCL1 and other chemokines. In the early stage of IRI, we found that S100a8/9 and CXCR2 were highly expressed in neutrophils, consistent with a previous study (30), but these cells were defined as CXCR2^+^ monocytes in another scRNA-Seq study (11). Furthermore, we identified Treml4_Monos as important monocytes that promoted inflammation and fibrosis during the progression from AKI to CKD (31, 32). Multiple studies have demonstrated that Treml4_Mono can activate the TLR4/7/NF-κB pathway and initiate the inflammatory response (33, 34). Last but not least, activated Fn1_Mac could further express chemokines such as *Ccl2* and *Ccl9* to recruit more immune cells as well as specifically express more THBS-1 to interact with *Cd36* of the surviving PTs, resulting in an irreversible inflammatory response. These findings suggest that targeted reduction of Fn1_Mac is an important therapeutic strategy for reducing the level of renal inflammation after IRI.

Tissue-derived EV are not only disease specific but can also directly affect the microenvironment of the damaged kidney in vivo. Plasma EV are a mixture of EV secreted by all organs, tissues, and cells in the body, but EV obtained directly from tissues through enzymatic hydrolysis have disease specificity (35, 36). Compared with EV isolated from cell culture in vitro, tissue-derived EV could better mimic the damaged microenvironment in vivo (35). For example, EV derived from HK2 cells were inadequate to simulate EV that originate from the proximal tubule in vivo. In addition, GW4869 significantly reduced the infiltration of MPs and immune inflammatory damage in IRI kidneys. Consistent with previous studies, GW4869, a noncompetitive inhibitor of N-Smase, regulates intercellular communication likely by blocking EV secretion, such as by mitigating the activation of myofibroblasts (37, 38) or by promoting the repair of renal tubule cells through markedly increasing EGFR activation to promote wound healing (39). Collectively, the potential mechanisms by which GW4869 might also play a key role in the resistance to PT injury in IRI, besides reducing immune cell infiltration, are interesting and necessary subjects to explore in the future. What’s more, the development of a method specifically targeted to inhibit the secretion of EV by injured renal tubular epithelial cells is very promising in regard to the reduction of the infiltration of immune cells and, thus, achieve the role of kidney protection.

To further examine the roles of kidney-EV, MS was used to analyze the proteins in IRI-EV. The results show unique protein signatures with obvious changes in integrin β1 and integrin α5, which are involved in a variety of BPs, especially cell adhesion and migration. Integrins are widespread transmembrane molecules that include α and β subunits, form the cell adhesion plaque complex, and affect cell adhesion and migration through dynamic assembly and dissociation (40). In addition, the MS results show that IRI-EV specifically contained THBS-1, which had been previously identified as a secretory factor mediating cell injury in IRI (41). Matricellular proteins belonging to the thrombospondin family are stress- and injury-induced mediators of cellular attachment dynamics and extracellular matrix protein production (42). Consistent with the existing studies, our protein molecular docking and co-IP results suggest that integrin β1 interacted with THBS-1 to mediate the adhesion and accumulation of immune cells, especially MPs (43, 44). Therefore, early blockade of tissue-derived EV release is a potentially novel idea to reduce the infiltration of immune cells during IRI.

To trace the origin of the EV protein, multiomics analysis of the scRNA-Seq and protein profiling data show that the ability of the PT-injured cells and the infiltrated MPs to secrete EV was significantly enhanced after IRI. Further analysis of protein function in EV revealed that the most significant difference in integrin β1 expression was in PT-injured cells, and *Thbs1* was highly expressed in Fn1_Mac, indicating a possible interaction between these 2 subsets of cells. Our previous studies show that integrins, specifically integrin β1, which have been identified on the surface of MSC/MSC-EV membranes, promoted their adhesion to extracellular matrix/biomaterial and migration to injury sites in IRI/cisplatin-induced AKI models (45, 46). The integrins on the surface of MSC-EV have also been shown to promote the delivery of these vesicles to the IRI injury site (21, 47). Moreover, integrin-rich EV have been shown to promote the adhesion, migration, and invasion of MPs in several disease processes (48–51). Ligand receptor analysis revealed that THBS-1 acted on PTs mainly through CD36 at the genetic level, which verified that CD36 acted as a coreceptor for TLR4/6 and promoted CXCL1 and IL-1β production by activating NF-κB and the NLRP3 inflammasome, amplifying the level of local inflammation in IRI kidneys (52, 53). Moreover, THBS-1 promoted renal fibrosis mainly by regulating the expression and activity of TGF-β in unilateral ureteral obstruction (UUO), a well-known model of renal fibrosis (54). However, THBS-1 receptor CD47/CD36 blockade by exogenous antibodies or gene KO in mice has therapeutic potential to prevent or suppress IRI-associated lesions (26, 55, 56). These findings suggest that THBS-1 and its receptors are potential targets to relieve inflammatory damage and renal fibrosis during AKI-CKD progression.

Of course, this study also had some limitations. The ratio for the 1:4 FACS indeed introduces artifacts into the single cell data because of the discrepancy between the number of cells that appear in the single cell analysis and the amount that appears in the tissue, as well as the discrepancy between transcription and EV protein packaging, which is not always a 1:1 relationship between gene and protein and which is finely tuned by specific activation of gene promoters (57), alternative splicing (58), translational reprogramming (59). Also, in terms of the methodology of gene and protein detection, gene detection is the acquisition of gene fragments through reverse transcription and then amplification, while protein mass spectra is a technique to detect the peptide mixture after the protein is digested by the protease. Thus, the bioinformatics analysis of each cell’s ability to produce EV in Figure 7B is not completely accurate, but it provides a possible research direction. This study only focused on the proteins in EV, but whether mRNA or noncoding RNAs, especially miRNAs, were also involved in the infiltration and activation of immune cells needs to be further studied.

In conclusion, a comprehensive multiomics analysis of scRNA-Seq data and the EV protein signature in IRI was performed to create an intercellular communication atlas at the single cell level and EV level in the IRI microenvironment. The IRI-PT–injured cells secreted chemokines and integrin-rich EV to recruit immune cells into the injured kidney and specifically activated Fn1 + Mac to amplify the local renal inflammatory level by expressing *Ccl2* to recruit more infiltrating MPs as well as by the interaction of THBS-1–CD36 to promote the secretion of IL-1β from surviving PTs. In conclusion, IRI-EV have a unique proteomic signature and a functional role in the regulation of MP migration and renal inflammation during IRI, providing an important therapeutic target.

## Methods

### Construction of the IRI model and blockade of EV production.

Male C57BL/6 mice (8 weeks old, 20–22 g body weight) were purchased from SPF Biotechnology. Bilateral IRI (BiIRI) was established by clamping the renal pedicle for 30 minutes, during which time the body temperature was strictly maintained at 37°C by a sensitive rectal probe (Harvard Apparatus), as previously described (60, 61). To block the secretion of EV, we administered 2.5 mg/kg GW4869 by i.p. injection 1 hour before IRI operation.

### Sample processing and scRNA-Seq.

The whole kidneys were minced and digested in 2 mL of Liberase (Roche) in RPMI-1640 medium at 37°C for 20 minutes. The resulting single-cell suspensions were filtered sequentially through 70 μm and 40 μm meshes before being incubated with FITC anti-CD45 (103108, BioLegend); cell viability was determined by exclusion of 7-aminoactinomycin (7-AAD, 00-6993-50, Thermo Fisher Scientific). These mixtures of living cells (CD45^+^/all cells = 1:4) sorted using the S3e Cell Sorter (Bio-Rad) were loaded onto a microfluidic chip by Berry Genomics, and a cDNA library was generated using the 10× Genomics platform (10× Genomics).

### Processing of scRNA-Seq data.

After production of gene expression matrices using the Cell Ranger count function, the Seurat R package (v4.0.3) was used to analyze subsequent data, including normalization, scaling, principal component analysis (PCA), UMAP dimension reduction, and visualization of gene expression (15, 62). Low-quality cells with < 500 or > 6,000 detected genes and cells with ≥ 25% mitochondria-related gene expression were filtered out. The double cells and batch effect were removed based on DoubletFinder (63) and Harmony (64), respectively. Cell clustering was conducted based on FindClusters (65). The FindVariableFeatures function was used to analyze the highly variable genes in the filtered cell data (65). Finally, RunUMAP was used to show the effect of cell clustering by using UMAP.

### Extraction of EV from kidney tissue and cell supernatants.

EV were extracted from kidney tissue as described previously with modifications (66). The dissociated enzyme mixture was resuspended according to the manufacturer’s instructions (Miltenyi Biotec). Kidney tissue (~100 mg) was weighed and sliced on dry ice before being incubated in the dissociation mixture for 15 minutes at 37°C.

The dissociated tissue or cell supernatant was filtered through a 70 μm filter twice to remove residual tissues. The suspension was centrifuged at 300*g* for 10 minutes and then at 2,000*g* for 10 minutes at 4°C. The cell-free supernatant was centrifuged at 10,000*g* for 20 minutes at 4°C and filtered through a 0.22 μm filter to further remove cell debris. The collected suspension was further processed by ultracentrifugation at 150,000*g* for 2 hours at 4°C. The obtained pellet was resuspended in 1 mL of phosphate-buffered saline (PBS) and further purified using Exosupur columns (Echobiotech). Finally, the fractions were concentrated to 200 μL through 100 kDa molecular weight cutoff Amicon Ultra spin filters (Merck) and stored at –80°C.

### Identification of EV.

The cup-shaped membrane structure of EV was identified by transmission electron microscopy (HT7700, Hitachi Ltd., Japan). The positive EV markers CD63 (1:400, sc-5275, Santa Cruz Biotechnology Inc.), HSP70 (1:1000, ab181606, Abcam), and TSG101 (1:1000, ab125011, Abcam) and the negative marker calnexin (1:750, 10427, Proteintech) were analyzed by Western blotting. Nanoparticle tracking analysis (NTA) was performed using a Zeta View PMX 110.

### MS.

Label-free liquid chromatograph-tandem MS (LC-MS/MS) detection was performed as previously described (67, 68). Briefly, total protein was extracted from purified kidney-EV. Then, the protein concentration was determined with a BSA standard protein solution curve. The obtained protein samples were digested with trypsin, eluted twice, and lyophilized. Finally, the separated peptides were analyzed by a Q Exactive HF-X mass spectrometer (Thermo Fisher Scientific) after sample elution. The raw LC-MS/MS data were searched in the UniProt database (http://www.uniprot.org). The identified protein contained at least 1 unique peptide with an FDR ≤ 0.01. In total, 2,602 proteins were selected for subsequent analyses. Gene Ontology (GO) enrichment analysis of the target proteins was implemented by the top GO R packages. Kyoto Encyclopedia of Genes and Genomes (KEGG) pathway enrichment analyses were conducted to annotate the pathway to understand the high-level functions and utilities of the biological system (http://www.genome.jp/kegg/). KOBAS software was used to test the statistical enrichment of differentially expressed genes in KEGG pathways.

### Combined analysis of scRNA-Seq data of kidney cells and LC-MS/MS data of kidney-EV.

The kidney-EV proteins from the LC-MS/MS data were specified by the selection criteria described in detail in a previous study (62). Specifically, the proteins detected in all samples of IRI group or sham group are screened; the protein expression level of the 3 samples in sham group or IRI group is not 0. The protein coding genes are also detected in the scRNA-Seq data of the corresponding samples. Then 1,777 proteins of sham group and 1,875 proteins of IRI group were selected for subsequent analyses. After the relatively highly expressed cluster markers were selected using the “Find All Markers” function in the Seurat package, the cluster markers of each cluster and the data on kidney-EV proteins were overlapped to classify the kidney-EV proteins as “cluster markers,” “noncluster markers,” or “not detected.” The formula for evaluating the ability of a single cell or a single cluster of cells to secrete EV has been described in detail in a previous study (62). The specific calculation method is as follows.

(Equation 1)
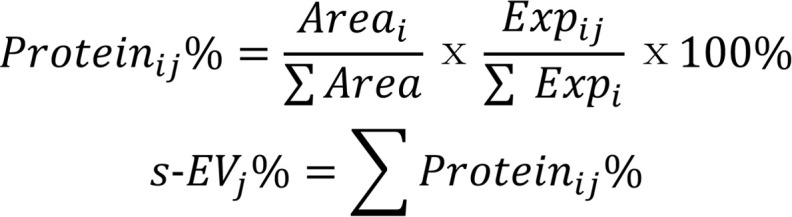


Thus, the EV secretion capacity of each cluster (t-EV%) is obtained by summing the s-EV% of all individual cells in the same cluster.

(Equation 2)
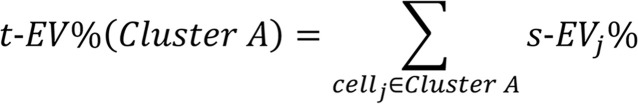


In these above formulas, Protein_ij_% represents the expression index of Protein_i_ in Cell_j_. Area_i_ represents the average peak area of Protein_i_ in the result of LC-MS/MS of kidney-EV, and Exp_ij_ represents the expression level of the gene that encodes Protein_i_ in Cell_j_ in the scRNA-Seq data of kidney cells. s-EV_j_% represents the EV secretion ability of Cell_j_.

### Gene set variation analysis (GSVA).

GSVA is a nonreference and unsupervised method for evaluating transcriptome data enrichment. The gene set was obtained via the MSigDB database (v7.5.1), and then each cell type was scored by evaluating changes in the pathway at the genetic level through GSVA software (v1.40.1), obtaining the coefficient of variation among cell types. Finally, the functional pathways with the top 50 coefficients of variation were selected to display the functional pathways with the most drastic changes among different cell types.

### Cell culture.

HK2 cells (human proximal tubular cells) and HPTCs were maintained in Dulbecco’s Modified Eagle Medium/Nutrient Mixture F-12 (DMEM/F12) medium (Thermo Fisher Scientific) with 10% FBS (Thermo Fisher Scientific) or 10% EV-free FBS (System Biosciences). After the HK2 cells were cultured with EV-free FBS for 48 hours, the culture supernatant was collected, and EV were extracted.

RAW264.7 cells (mouse leukemia monocyte/macrophage line) were cultured in RPMI 1640 medium (Thermo Fisher Scientific) supplemented with 10% FBS with/without EV derived from renal tissue dissociation solution.

293T cells (human embryonic kidney cells) were cultured in DMEM supplemented with 10% FBS.

These cells were purchased from ATCC and were grown under standard conditions (37°C, 5% CO_2_), and the medium of these cells was routinely replaced every other day.

### Establishment of the in vitro hypoxia model.

To mimic IRI in vitro, HK2 cells were cultured in a humidified hypoxia/reoxygenation (H/R) chamber (1% O_2_, 5% CO_2_, and 94% N_2_, 37°C) in DMEM without FBS for 12 hours and then returned to a reoxygenated environment (5% CO_2_ and 21% O_2_, 37°C) in DMEM with 10% FBS.

### Tracking EV with Dil staining.

EV were first labeled with 1,1′-dioctadecyl-3,3,3′,3′-tetramethylindocarbocyanine perchlorate (Dil) and then added to the medium of RAW264.7 cells for 8 hours. Laser scanning confocal microscopy or fluorescence microscopy was used to determine whether the EV could be internalized.

### In vitro macrophage stimulation assay.

For the macrophage stimulation assay, 20 μg/mL EV isolated from sham and IRI kidneys were added to RAW264.7 cells and cultured for 24 or 48 hours, after which the cells were harvested for RNA analysis and cell migration assays.

### Cell migration assay.

A Transwell migration chamber (Corning, 8.0 μm) was used to determine the migratory abilities of MPs. RAW264.7 cells were inoculated in the upper chamber, and HK2 cells were inoculated in the lower chamber. Prior to being cocultured with RAW264.7 cells, HK2 cells were subjected to hypoxia for 12 hours and subsequently stimulated with or without 20 μM GW4869 (Sigma-Aldrich) during reoxygenation. After 48 hours of coculture, the cells in the upper chambers were removed, fixed with 4% paraformaldehyde, and stained with 0.1% crystal violet (Beyotime) for 15 minutes. After 3 washes with PBS, cell migration was examined by ordinary light microscopy.

### Cell transfection with siRNA.

HK2 cells were transfected with ITGB1 siRNA or negative control (NC) siRNA (Gene Pharma) using Lipofectamine 3000 (Invitrogen, USA). The sequences were as follows: *ITGB1*, sense: 5′-GGCUCCAAAGAUAUAAAGATT-3′, antisense: 5′-UCUUUAUAUCUUUGGAGCCTT -3′; *NC*, sense: 5′- UUCUCCGAACGUGUCACGUTT-3′, antisense: 5′-ACGUGACACGUUCGGAGAATT-3′.

### Histopathological examination.

For histopathological examinations, 2 μm–thick kidney sections were stained with PAS and H&E according to standard protocols. The ATN scores of PAS-stained sections were determined as follows: 0, no damage; 1, involvement of < 25% of the damage area; 2, 25%–50% of the damage area; 3, 50%–75% of the damage area; and 4, > 75% of the damage area.

### IHC and immunofluorescence staining.

IHC or immunofluorescence staining was performed as described previously (69). The primary antibodies used were PCNA (1:16,000, 13110, Cell Signaling Technology [CST]), CD68 (1:200, GB113886, Servicebio), IL-17a (1:400, GB11110-1-100, Servicebio), CXCL1 (1: 500, 12335-1-AP, Proteintech), TGF-β1 (1: 500, ab215715, Abcam), THBS-1 (1: 5,000, ab267388, Abcam), integrin β1 (1: 500, 26918-1-AP, Proteintech), and C3 (1: 2,000, 21337-1-AP, Proteintech).

### Western blotting and co-IP.

Samples of kidneys, cells, and EV were homogenized in radioimmunoprecipitation assay (RIPA) lysis buffer (Thermo Fisher Scientific) supplemented with phenylmethylsulfonyl fluoride and phosphatase inhibitors (Beyotime). The obtained proteins were quantified by a BCA protein assay kit (Thermo Fisher Scientific). Equal amounts of the denatured protein samples (~20 μg/lane) were separated by sodium dodecyl sulfate‒polyacrylamide gel electrophoresis (SDS-PAGE) and were then transferred to nitrocellulose filter membranes. The membranes were incubated with primary antibodies at 4°C overnight. For co-IP, 293T cells were transfected with Flag-labeled *Thbs1* plasmids and Myc-labeled *Itgb1* plasmids for 48 hours. The obtained cell lysates were precleaned and incubated with anti-Myc or anti-Flag magnetic beads (Thermo Fisher Scientific). The captured proteins were eluted and subjected to Western blotting with anti-Flag or anti-Myc antibodies. The primary antibodies were as follows: anti–THBS-1 (ab267388), anti–TNF-α (ab66579), anti–IL-1β (ab283818), and anti–IL-6 (ab259341, 1:1000) from Abcam; anti–pi–NF-κB (3033) and anti-GAPDH (2118, 1:1000) from CST; anti–KIM-1 (1:5,000, AF1817) from R&D Systems; and anti–integrin β1 (26918-1-AP), anti–integrin α5 (10569-1-A, 1:1,000), anti-GAPDH (1:2,000), and anti–β-actin (1:10,000) from Proteintech. The blots were detected using chemiluminescence and autoradiography (Bio-Rad). Finally, semiquantitative analysis of the blots was performed using ImageJ (NIH).

### Detection of mRNA.

Total RNA was extracted using TRIzol reagent (Invitrogen) according to the manufacturer’s protocol. The RNA was quantified using a NanoDrop1000. cDNA was transcribed using PrimeScript reverse transcription reagent (Bio-Rad). A real-time PCR kit (Takara) was used on an iCycler (Bio-Rad) to quantify the mRNA. The following primer sequences were used for the following genes: *mCcl2*, 5′-TTAAAAACCTGGATCGGAACCAA-3′ (forward), 5′-GCATTAGCTTCAGATTTACGGGT-3′ (reverse); *mCd86*, 5′-TGTTTCCGTGGAGACGCA AG-3′ (forward), 5′-TTGAGCCTTTGTAAATGGGCA-3′(reverse); *mTnfa*, 5′-CCCTCACACT CAGATCATCTTCT-3′ (forward), 5′-GCTACGACGTGGGCTACAG-3′ (reverse); *mIl1b*, 5′-GCAACTGTTCCTGAACTCAACT-3′ (forward), 5′-ATCTTTTGGGGTCCGTCAACT-3′ (reverse); *hIl1b*, 5′- TTCGACACATGGGATAACGAGG-3′ (forward), 5′- TTTTTGCTGTG AGTCCCGGAG-3′ (reverse); *mActb*, 5′-ACTGCCGCATCCTCTTCCT-3′ (forward), 5′-TCAA CGTCACACTTCATGATG-3′ (reverse); and *hGapdh*, 5′- ACAACTTTGGTATCGTGGAAGG-3′ (forward), 5′-GCCATCACGCCACAGTTTC-3′ (reverse).

### Flow cytometry.

After the kidney tissue was dissociated by Liberase TM (Roche) in RPMI-1640 medium at 37°C for 20 minutes, 100 μL of the single-cell suspensions was incubated with the corresponding antibodies at room temperature for 30 minutes in the dark. The following antibodies were purchased from BioLegend: FITC anti-CD45 (catalog 103108), APC/Cyanine7 anti-CD11b (catalog 101226), PE/Cyanine7 anti-F4/80 (catalog 123114), and APC anti-Ly6c2 (catalog 128016). PE anti-Arg-1 (catalog IC5868P) was purchased from R&D Systems. The data were analyzed by using Kaluza.

### Molecular docking.

All protein structures were modeled in the Molecular Manipulation Environment (MOE 2019.1) platform. HDOCK software was used to set the protein as rigid and the docking contact site as a full surface, and the conformations generated after docking were set to 100. The most negative conformations were selected by using the scoring function and visualized by PyMOL 2.1 software.

### Cytokine measurement.

The levels of THBS-1 in the IRI kidney were measured using ELISA kits (USCN Life Science).

### Apoptosis analysis.

A TUNEL (Beyotime) assay was used to detect apoptosis in paraffin sections of mouse kidney tissue.

### Statistics.

Measurement data are expressed as the mean ± SD. Two-tailed Student’s *t* tests were used to compare 2 groups. One-way ANOVA was performed to compare 3 or more groups. All analyses were conducted with GraphPad Prism software. *P* < 0.05 was considered significant.

### Study approval.

The animal care and experimental procedures were approved by the ethics committees for animal experimentation of Chinese People’s Liberation Army (PLA) General Hospital (no. 2022-X18-30).

### Data availability.

The data and materials that support the findings of this study are available from the corresponding author upon reasonable request. The raw data reported in this paper have been deposited on OMIX (accession no. 004421). The R package source code is available on GitHub (wwwwenjuan/Analysis-of-kidney-scRNA-Sequencing; commit ID: 166c058). Values for all data points in graphs are reported in the Supporting Data Values file.

## Author contributions

GC, QH, and WW conceived and designed the experiments. WW and XR conducted the experiments. WW performed the data analysis. GC, QH, and XC provided valuable advice and were responsible for research supervision, coordination, and strategy. WW drafted the manuscript. GC and QH reviewed and edited the manuscript. All authors read and approved the final manuscript.

## Supplementary Material

Supplemental data

Supporting data values

## Figures and Tables

**Table 1:**
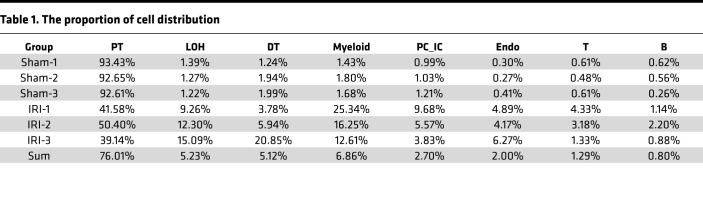
The proportion of cell distribution

## Version 1 01/23/2024

Electronic publication
