# Supplementary material for: Integrin β1–rich extracellular vesicles of kidney recruit Fn1+ macrophages to aggravate ischemia-reperfusion–induced inflammation
Source: JCI Insight. 2024 Jan 23;9(2):e169885. doi: 10.1172/jci.insight.169885 (PMC10906229; doi:10.1172/jci.insight.169885)
Supplement: Supplemental data [file jciinsight-9-169885-s221.pdf]

Supplemental figures：

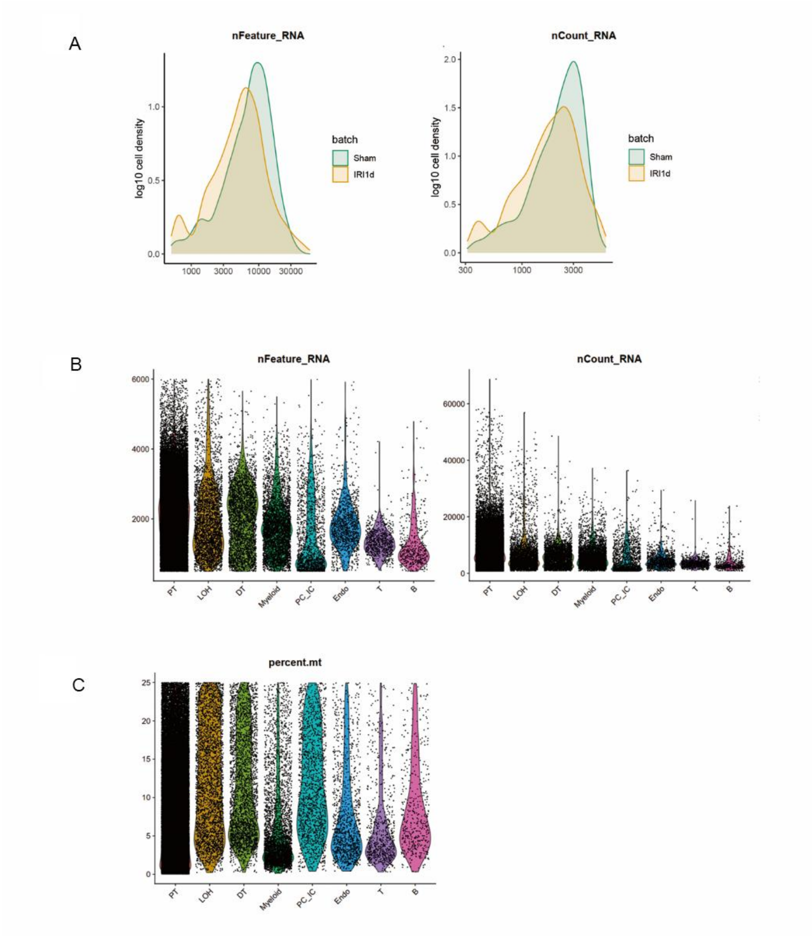

Figure S1 Data quality control and filtering criteria.

(A) Distribution of detected gene numbers and unique molecular identifier (UMI) counts in sham, IRI1d kidneys. (B) The distribution of detected gene numbers and UMI counts in different cell types. (C) Filtering criteria of for mitochondrial proportion.

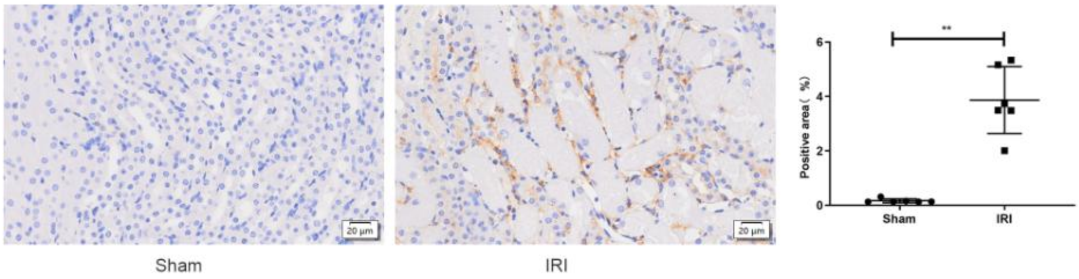

Figure S2 Immunostaining and semi-quantitative analysis showing the level of THBS-1 in sham group and IRI group.

A

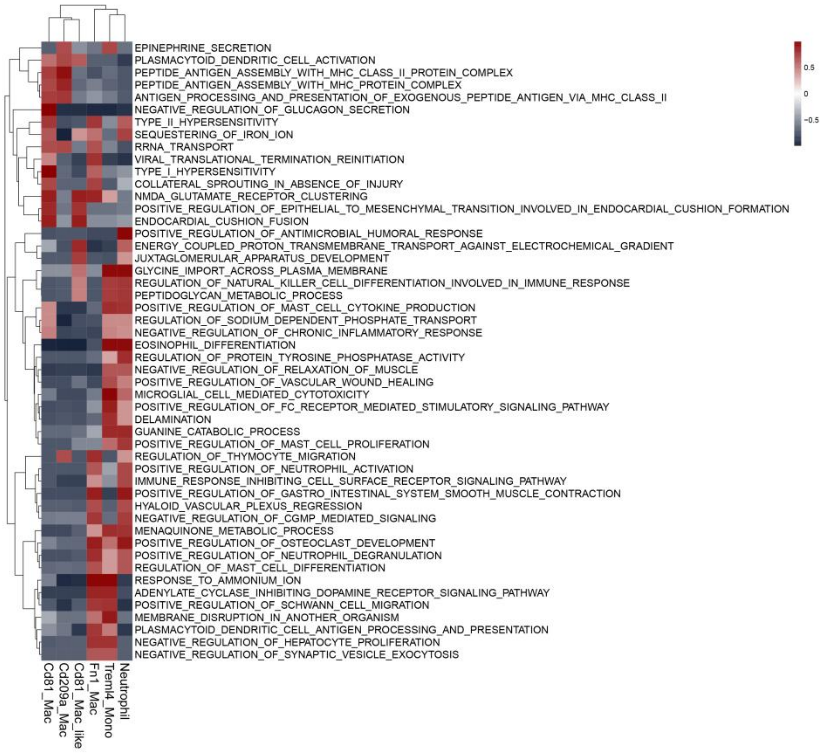

B

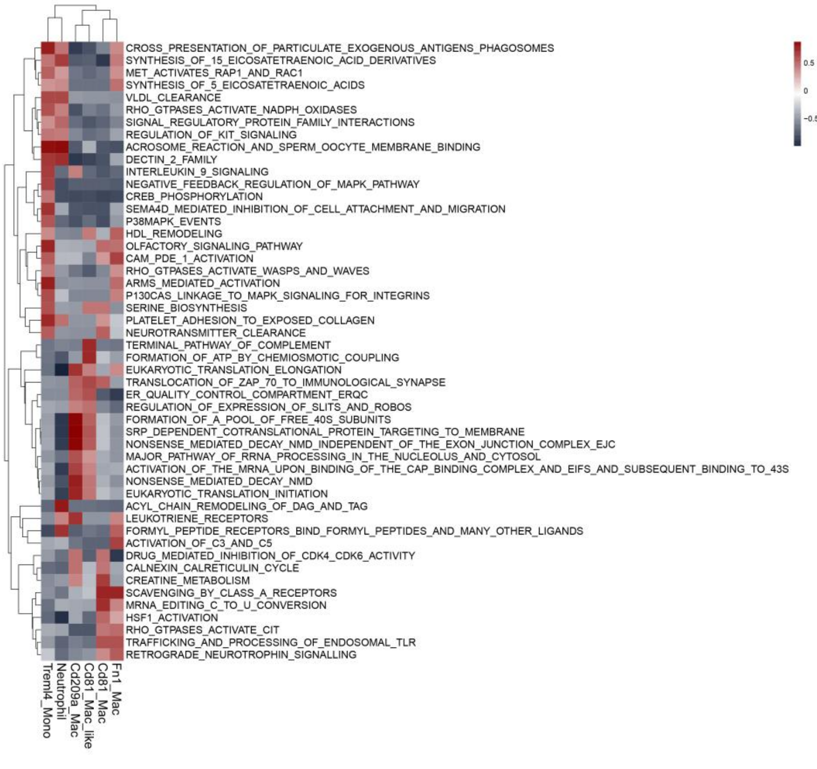

Figure S3 The biological process (BP, A) and reactome analysis (B) showing the most obvious changes in the top 50 functions and pathways between the different clusters. Red represents pathway activation; blue represents pathway inhibition.

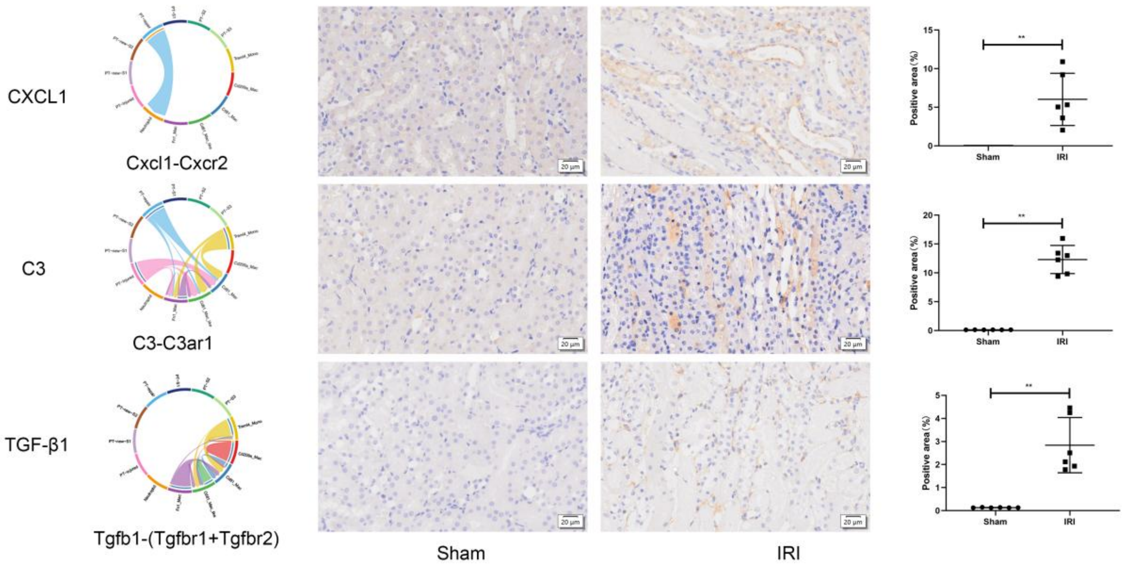

Figure S4. Chord diagram and immunostaining analysis showing the myeloid cell-PT immune interaction networks.

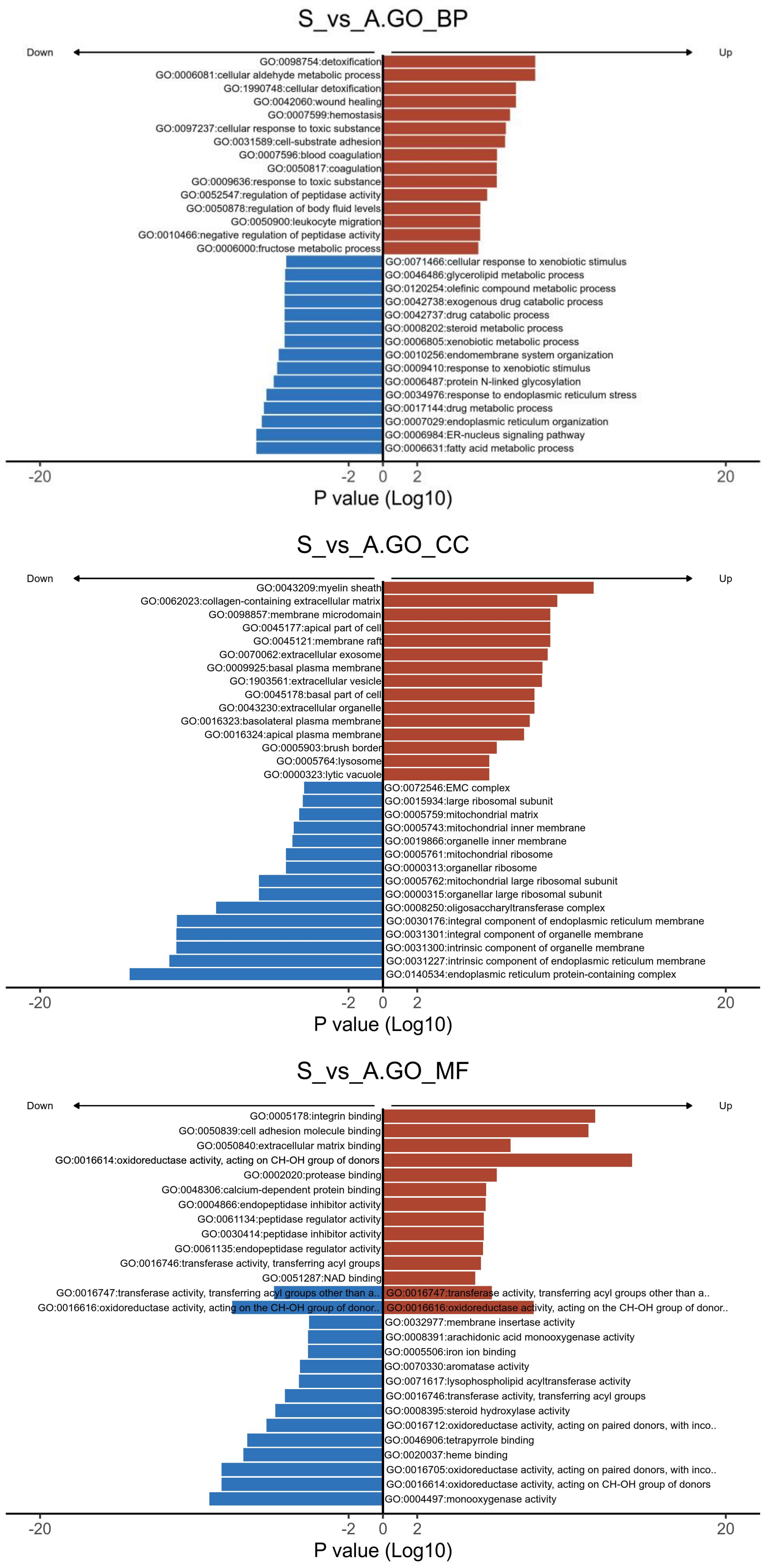

Figure S5. Enriched Gene Ontology (GO) term analyses of differential proteins in EV.

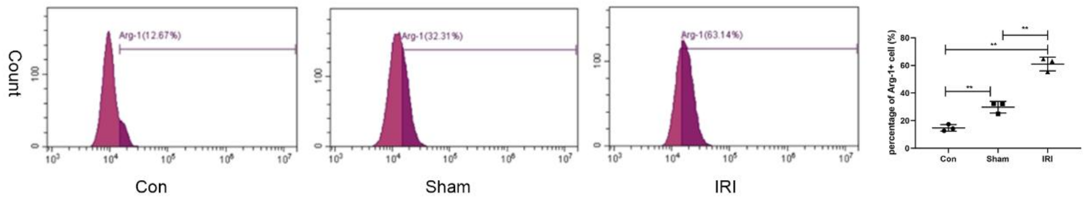

Figure S6 The flow cytometry detection of RAW264.7 cells differentiation in the kidney tissue microenvironment of sham and IRI.

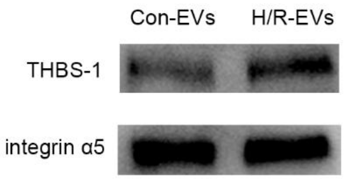

Figure S7 Western blotting showing the production of THBS-1 and integrin α5 in con-EVs and H/R-EVs of HK2 cells.

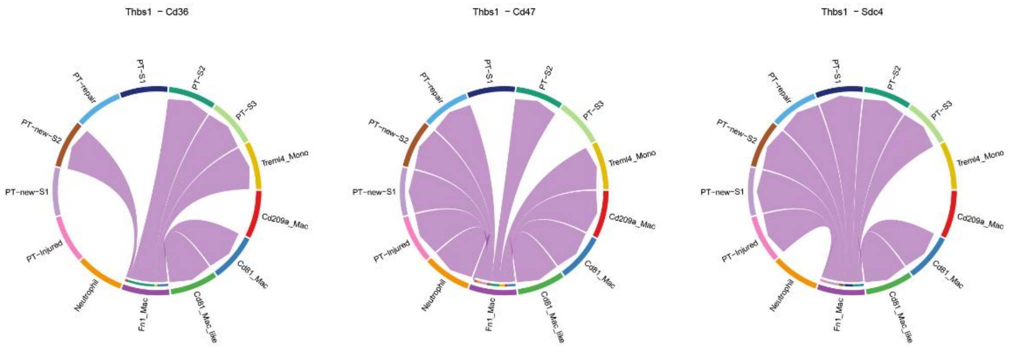

Figure S8 Chord diagram demonstrating potentially intercellular interactions via *Thbs1* and its ligands at the gene level.

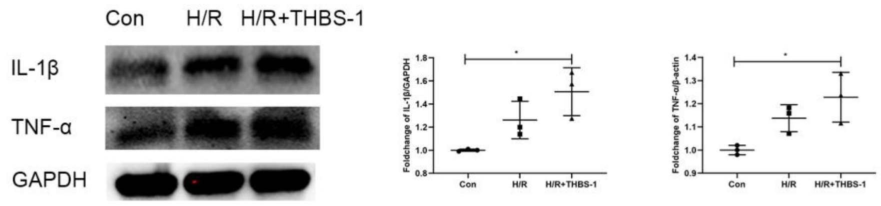

Figure S9 Western blotting and semi-quantitative analysis showing the role of THBS-1 in the expression of IL-1β and TNF-α in human proximal renal tubular epithelial cells.
